# Pulmonary Embolism as a Rare Complication of Anaplasmosis: A Case Report

**DOI:** 10.7759/cureus.102307

**Published:** 2026-01-26

**Authors:** Nabil Varwani, Arshia Ahmed, Salman J Khan, Lela Adeoshun

**Affiliations:** 1 Internal Medicine, Guthrie Lourdes Hospital, Binghamton, USA; 2 Public Health, University of Massachusetts, Amherst, USA

**Keywords:** anaplasma phagocytophilum, anaplasmosis, antiphospholipid antibodies, pulmonary embolism, tick-borne disease, venous thromboembolism

## Abstract

Pulmonary embolism is an exceptionally rare complication of anaplasmosis. We report a case of a 69-year-old man with acute *Anaplasma phagocytophilum* infection who developed bilateral pulmonary emboli and pneumonia despite appropriate doxycycline therapy. Evaluation revealed positive antiphospholipid antibodies, raising concern for antiphospholipid syndrome; however, interpretation was confounded by acute infection and ongoing anticoagulation, limiting the ability to distinguish transient infection-associated antibody positivity from an underlying thrombophilic disorder. This case highlights the need to consider venous thromboembolism in patients with anaplasmosis presenting with chest pain or respiratory symptoms and underscores the diagnostic uncertainty surrounding antiphospholipid antibody testing in the setting of acute infection and thrombosis.

## Introduction

Anaplasmosis is an increasingly recognized tick-borne infection in the upper Midwest and northeastern United States, transmitted by *Ixodes* ticks, which also serve as the vector for Lyme disease. The incidence of tick-borne illnesses in the United States has risen steadily over the past two decades, largely due to expanding tick habitats, climate change, and increased human exposure. *Anaplasma phagocytophilum*, the causative organism, is an obligate intracellular bacterium that infects neutrophils [1]. Patients commonly present with non-specific symptoms, such as fever, headache, myalgias, and malaise, which often lead to diagnostic uncertainty. In this context, careful attention to epidemiologic factors, including recent travel to endemic regions, outdoor activities such as hiking or camping, and known or potential tick exposure, is critical to prompt recognition and diagnosis. Characteristic laboratory abnormalities, including thrombocytopenia, leukopenia, and elevated transaminases, further support the diagnosis when interpreted alongside clinical and exposure history [2].

Most cases respond promptly to doxycycline and resolve without long-term complications [3]. However, severe disease has been reported, with complications such as renal failure, pneumonia, and coagulopathy [4,5]. Thrombotic manifestations are exceedingly rare, with only isolated reports of events such as cerebral infarction [6]. Pulmonary involvement in anaplasmosis typically manifests as pneumonitis or, in severe cases, acute respiratory distress syndrome [1,2]. However, pulmonary embolism has not been previously reported as a complication of acute anaplasmosis, making such a presentation exceptionally rare.

We report a case of anaplasmosis complicated by pulmonary embolism, an uncommon thrombotic manifestation that expands the clinical spectrum of this infection. This case also underscores the diagnostic challenge posed by transient antiphospholipid antibody positivity during acute infection, which may mimic antiphospholipid syndrome (APS) and complicate decisions regarding thrombophilia evaluation and long-term anticoagulation.

## Case presentation

A 69-year-old man with a past medical history significant for recurrent nephrolithiasis, primary hyperparathyroidism, and vitamin D deficiency presented to the emergency department (ED) with four days of progressive, sharp, pleuritic pain in his left flank and upper abdomen. He denied dyspnea, cough, hemoptysis, or lower extremity swelling. There was no history of recent travel, immobilization, or trauma.

Two weeks prior to presentation, the patient had sustained a tick bite. About one week later (eight days before coming to the ED), he developed fever (102°F), myalgias, and malaise and was seen at an urgent care clinic. At that visit, he was hemodynamically stable with an unremarkable cardiovascular and pulmonary exam. Given his presentation and tick exposure history, he was empirically started on doxycycline 100 mg twice daily, and a tick-borne disease panel was ordered. Testing subsequently returned positive for *A. phagocytophilum*, and he continued doxycycline therapy. The patient had completed six days of doxycycline prior to presentation.

On examination in the ED, vital signs were within normal limits with normal oxygen saturation on room air. His BMI was 25 kg/m². Other than mild tachycardia, the cardiovascular and pulmonary exams were otherwise unremarkable. Abdominal exam showed tenderness to palpation in the left upper quadrant and flank without rebound or guarding. Costovertebral angle (CVA) tenderness was absent. Lower extremity examination was unremarkable, with no edema, erythema, or calf tenderness. A 12-lead electrocardiogram revealed a normal sinus rhythm with normal intervals, no acute ischemic changes, and no evidence of right ventricular strain pattern.

Lab work showed a marked inflammatory response with leukocytosis (white blood cell (WBC) 20.37 × 10³/µL), elevated CRP (17.0 mg/dL; normal range 0.00-0.50 mg/dL), and elevated liver enzymes (aspartate aminotransferase 20 U/L, alanine aminotransferase 72 U/L)-notably improved from initial values of aspartate aminotransferase 138 U/L and alanine aminotransferase 143 U/L (Table 1). Urinalysis showed no evidence of UTI. Lactate levels were normal throughout admission.

**Table 1:** Key laboratory findings on initial urgent care presentation, hospital admission, and follow-up WBC: white blood cell; AST: aspartate aminotransferase; ALT: alanine aminotransferase

Parameter	Initial urgent care visit	Admission	Follow-up (~3 months)	Reference range
WBC	6.02 K/µL	20.37 K/µL	8.60 K/µL	4.23-9.07 K/µL
Platelets	140 K/µL	391 K/µL	318 K/µL	163-337 K/µL
AST	138 U/L	20 U/L	17 U/L	<40 U/L
ALT	143 U/L	72 U/L	11 U/L	<50 U/L
Antithrombin III antigen	—	76%	100%	80%-120%

**Figure 1:**
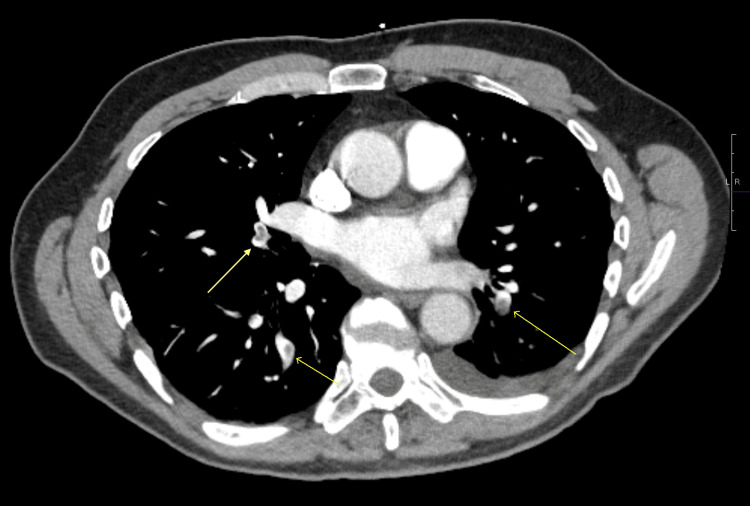
CTPA showing bilateral pulmonary emboli CTPA: CT pulmonary angiography

**Figure 2:**
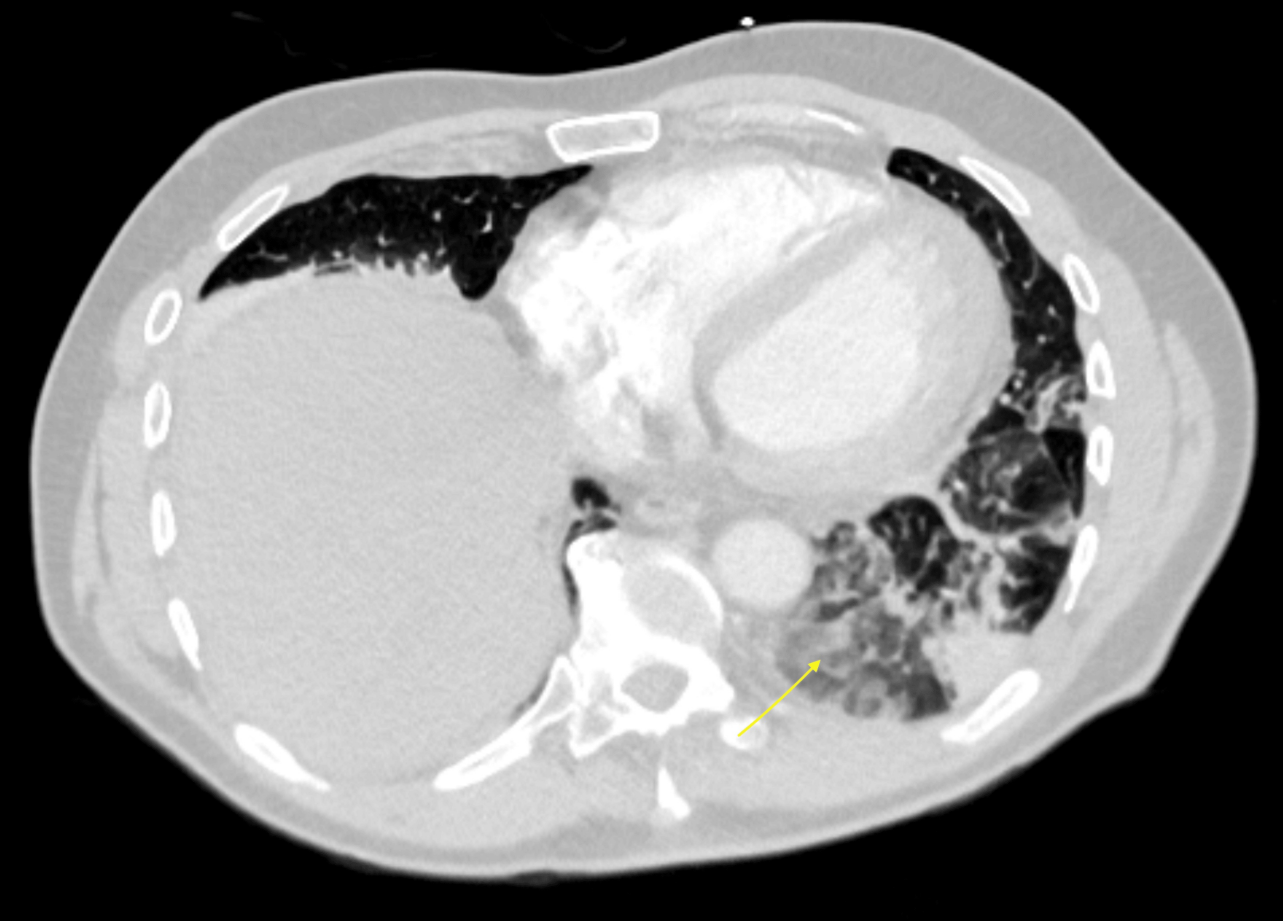
Patchy consolidation in bilateral lower lobes more so on the left

CT of the abdomen and pelvis without intravenous (IV) contrast showed patchy bibasilar airspace opacities, a small left pleural effusion, and bilateral non-obstructing renal calculi. Because of persistent pleuritic chest pain, we obtained a CT pulmonary angiogram, which revealed acute bilateral pulmonary emboli in the segmental and subsegmental branches of both lower lobes (Figure 1), along with bibasilar consolidations interpreted as suspected bibasilar pneumonia (Figure 2). A transthoracic echocardiography was performed and revealed a normal left ventricular ejection fraction (LVEF 65%-70%) with a normal diastolic function. Right atrial pressure was elevated at 15 mmHg (normal <5 mmHg). Pulmonary artery peak systolic pressure was 31 mmHg (normal <30 mmHg, mildly elevated). Additional findings included mild aortic valve sclerosis and mild tricuspid regurgitation.

The patient was admitted and started on anticoagulation with IV unfractionated heparin. The bibasilar infiltrates and leukocytosis were concerning for superimposed bacterial pneumonia, so we added ceftriaxone 2 g IV daily to provide empiric coverage for community-acquired pneumonia while he continued doxycycline to complete a 10-day course.

The patient had no traditional risk factors for venous thromboembolism (VTE). He was not taking any medications and had no personal or family history of clotting disorders or autoimmune disease. His surgical history included foot and hand surgeries, hernia repair, and lithotripsy, performed more than 12 months prior to presentation, none of which were complicated by VTE or prolonged immobilization. He denied a personal history of malignancy. Family history was notable for ovarian cancer in his mother, pituitary tumor in his father, skin cancer in his sister, and cancer of unknown type in his maternal grandmother.

The patient's clinical condition improved steadily during his four-day hospitalization with continued anticoagulation and antibiotics. His pleuritic chest pain improved, and inflammatory markers normalized. Oral anticoagulant options were discussed with the patient, and he was discharged on rivaroxaban 15 mg twice daily for three weeks, followed by 20 mg daily, with outpatient hematology follow-up.

Because of the unprovoked nature of his VTE, a comprehensive hypercoagulable workup (Table 2) was performed during hospitalization. Results came back after the patient got discharged, showing positive lupus anticoagulant, positive beta-2 glycoprotein I IgG antibodies, and weakly positive cardiolipin IgG antibodies. Protein C and protein S were assessed by both antigen levels and activity levels (functional assay), and both were normal. Antiphosphatidylserine antibodies and prothrombin gene mutation (G20210A) were all negative. Antithrombin III level was mildly decreased at 76% (normal range 80%-120%), likely related to heparin therapy. Repeat antiphospholipid antibody testing was scheduled at 12 weeks per APS diagnostic criteria.

**Table 2:** Inpatient hypercoagulable panel Ab: antibody

Test (during hospital admission)	Result	Reference range
Lupus anticoagulant (ratio)	1.65	<1.14 (negative)
Beta-2 glycoprotein I Ab (IgG)	12 U/mL	<7 negative, 7-10 equivocal, >10 positive
Beta-2 glycoprotein I Ab (IgM)	1 U/mL	<7 negative, 7-10 equivocal, >10 positive
Cardiolipin Ab (IgG)	13 U/mL	<10 negative, 10-40 weakly positive, >40 positive
Cardiolipin Ab (IgM)	4.8 U/mL	<10 negative, 10-40 weakly positive, >40 positive
Protein C	Normal	Normal
Protein S	Normal	Normal
Phosphatidylserine Ab	Negative	Negative
Factor II G20210A mutation	Negative	Negative
Factor V Leiden mutation	Negative	Negative
Antithrombin III antigen	76%	80%-120%

At his three-month hematology follow-up, the patient remained asymptomatic with complete resolution of chest pain and had returned to his baseline functional status. He reported no bleeding or thrombotic complications. Physical exam was unremarkable, and repeat labs (Table 1) showed normalization of inflammatory markers, liver enzymes, and antithrombin III levels.

Repeat hypercoagulable testing was performed while the patient was still on rivaroxaban 20 mg daily. Testing revealed persistent lupus anticoagulant (ratio 1.27, down from 1.65 acutely), mildly elevated beta-2 glycoprotein I IgG, cardiolipin IgG, beta-2 glycoprotein I IgA, and cardiolipin IgA antibodies (Table 3).

**Table 3:** Follow-up antiphospholipid panel (~3 months post-discharge) Ab: antibody

Test (after 3 months)	Result	Status	Reference range
Lupus anticoagulant (ratio)	1.27	High	<1.14 (negative)
Beta-2 glycoprotein Ab (IgG)	15.0 U/mL	Positive	<7 negative, 7-10 equivocal, >10 positive
Beta-2 glycoprotein Ab (IgA)	8.6 U/mL	Equivocal	<7 negative, 7-10 equivocal, >10 positive
Beta-2 glycoprotein Ab (IgM)	<2.4 U/mL	Negative	<7 negative, 7-10 equivocal, >10 positive
Cardiolipin Ab (IgG)	17.0 U/mL	Equivocal	<10 negative, 10-40 weakly positive, >40 positive
Cardiolipin Ab (IgA)	17.0 U/mL	Equivocal	<14 negative, 14-20 equivocal, >20 positive
Cardiolipin Ab (IgM)	3.4 U/mL	Negative	<10 negative, 10-40 weakly positive, >40 positive
Antithrombin III antigen	100%	Normal	80%-120%

The persistence of antiphospholipid antibodies at 12 weeks raised concern for possible APS. However, the confounding effect of rivaroxaban on lupus anticoagulant testing, the only mildly elevated/equivocal antibody titers, and the acute infection context made it difficult to reach a definitive diagnosis. Further testing after discontinuation of anticoagulation may be necessary to clarify whether this represents true APS. Recommendation on switching to warfarin was given to the patient, which is preferred over direct oral anticoagulants (DOACs) for APS. The patient, however, declined due to monitoring requirements. He opted to continue with rivaroxaban, understanding the risks involved.

## Discussion

Anaplasmosis remains an important tick-borne infection in endemic areas. While most cases are straightforward to diagnose and treat, this case shows that serious complications can occur, even in patients receiving appropriate therapy [1,2].

*A. phagocytophilum* infects neutrophils, triggering a substantial inflammatory response with multiple cytokines including interferon-γ, interleukin-10, interleukin-12 p70, and tumor necrosis factor-α [7]. The inflammatory cascade compromises the neutrophil's antimicrobial capacity, explaining the clinical manifestations of anaplasmosis-pancytopenia, liver dysfunction, and, in severe cases, shock and organ failure [8]. However, the same inflammatory mechanisms can create a prothrombotic state through endothelial activation, tissue factor exposure, and promotion of platelet adhesion, shifting the coagulation balance toward clot formation [9]. In susceptible individuals, this inflammatory stimulus suffices to trigger significant thrombosis. While there are case reports documenting various thrombotic complications in severe anaplasmosis, including arterial thrombosis [6] and severe coagulopathy [1,3,5], pulmonary embolism remains exceptionally rare.

The presence of positive antiphospholipid antibodies in our patient added diagnostic complexity. Infections are well-established triggers for transient antiphospholipid antibodies [10]. These infection-associated antibodies typically differ from those in primary APS-they tend to be lower titer, may target different epitopes, and usually disappear within weeks to months after the infection resolution. However, some patients with apparent infection-triggered antibodies are ultimately found to have underlying APS that was unmasked by the infection. Distinguishing between these scenarios requires repeat testing at 12 weeks, per the Sydney criteria for APS diagnosis [11].

An important limitation in our case was the timing of repeat testing. The 12-week antiphospholipid panel was obtained while the patient was still on rivaroxaban, which interferes with lupus anticoagulant assays. Rivaroxaban prolongs the dilute Russell viper venom time (the assay used for lupus anticoagulant detection), leading to false-positive results [12,13]. However, it does not typically interfere with enzyme-linked immunosorbent assay (ELISA)-based antibody measurements [13]. The ideal approach would have been to either test before initiating anticoagulation (not feasible in a patient with acute pulmonary embolism) or to wait after a washout period [12].

The diagnosis remains uncertain. The lupus anticoagulant result cannot be reliably interpreted, given rivaroxaban interference. The anticardiolipin IgG and beta-2 glycoprotein I IgG titers, while unaffected by rivaroxaban, are only mildly elevated or equivocal, lower than typically seen in primary APS. The initial antithrombin III reduction (76%) likely reflected heparin-induced consumption rather than inherited deficiency, as evidenced by normalization to 100% at three-month follow-up. Our patient may have transient antibodies that will eventually disappear, or he may have true APS. Continued anticoagulation seems reasonable given his unprovoked pulmonary embolism. Should anticoagulation discontinuation be considered in the future, repeat testing off medication would be helpful to clarify the diagnosis.

An additional consideration is anticoagulant choice. Warfarin remains the preferred agent for APS-associated thrombosis, as DOACs have shown inferior efficacy in this population. Our patient declined warfarin due to monitoring requirements and elected to continue rivaroxaban. While this is reasonable for unprovoked VTE of unclear etiology, it may be suboptimal if true APS is confirmed in the future.

This case illustrates several important clinical points. First, although rare, thrombotic complications should be on your differential in patients with anaplasmosis who develop respiratory symptoms or chest pain. The inflammatory state induced by *A. phagocytophilum* can precipitate thrombosis in susceptible individuals. Second, timing is critical when evaluating hypercoagulable states in the setting of acute infection. Ideally, testing should be deferred until the infection has resolved and the patient is off anticoagulation, but clinical circumstances do not always allow for ideal timing. Third, the intersection of infection, inflammation, and coagulation is more complex than we appreciate in routine practice. Infections can both cause transient antibodies and unmask underlying thrombophilias [14].

Long-term management will require ongoing assessment. If his clinical course remains uncomplicated and anticoagulation discontinuation is eventually considered, repeat testing off medication would clarify the diagnosis.

## Conclusions

We present a rare case of bilateral pulmonary embolism complicating acute anaplasmosis. This case emphasizes that tick-borne illnesses can present with complications beyond their typical clinical patterns. It stresses the value of comprehensive evaluation and careful follow-up in patients with unprovoked thrombotic events, particularly when infection and coagulation disorders intersect, creating diagnostic uncertainty. While anaplasmosis usually follows a benign course with doxycycline therapy, it can occasionally induce a prothrombotic state through inflammatory and endothelial activation mechanisms. Clinicians should maintain a high index of suspicion for VTE when patients with anaplasmosis develop respiratory symptoms or pleuritic chest pain.
